# Supplementary material for: Heparin-based hydrogel scaffolding alters the transcriptomic profile and increases the chemoresistance of MDA-MB-231 triple-negative breast cancer cells
Source: Biomater Sci. 2020 Feb 13;8(10):2786–96. doi: 10.1039/c9bm01481k (PMC7497406; doi:10.1039/c9bm01481k)
Supplement: Supplementary file 2 [file BM-008-C9BM01481K-s002.zip › Supplementary File 4/HP-B hydrogel + EGF (s)vControl/Pathways/my_analysis.Gsea.1545201170987/HALLMARK_GLYCOLYSIS.html]

Details for gene set HALLMARK\_GLYCOLYSIS[GSEA]

|  || Dataset | expr.class.cls#HYDEGF\_versus\_CONTROL.class.cls#HYDEGF\_versus\_CONTROL\_repos |
| Phenotype | class.cls#HYDEGF\_versus\_CONTROL\_repos |
| Upregulated in class | CONTROL |
| GeneSet | HALLMARK\_GLYCOLYSIS |
| Enrichment Score (ES) | -0.4511095 |
| Normalized Enrichment Score (NES) | -2.135483 |
| Nominal p-value | 0.0 |
| FDR q-value | 0.0 |
| FWER p-Value | 0.0 |
Table: GSEA Results Summary

  

Fig 1: Enrichment plot: HALLMARK\_GLYCOLYSIS      
 Profile of the Running ES Score & Positions of GeneSet Members on the Rank Ordered List

  

| PROBE | DESCRIPTION (from dataset) | GENE SYMBOL | GENE\_TITLE | RANK IN GENE LIST | RANK METRIC SCORE | RUNNING ES | CORE ENRICHMENT || 1 | PHKA2 | na |  |  | 740 | 1.873 | -0.0262 | No |
| 2 | EXT1 | na |  |  | 1070 | 1.642 | -0.0323 | No |
| 3 | ADORA2B | na |  |  | 1353 | 1.493 | -0.0370 | No |
| 4 | EGFR | na |  |  | 1395 | 1.470 | -0.0291 | No |
| 5 | GMPPB | na |  |  | 1436 | 1.455 | -0.0213 | No |
| 6 | SLC25A10 | na |  |  | 1736 | 1.346 | -0.0279 | No |
| 7 | CHST12 | na |  |  | 2738 | 1.068 | -0.0732 | No |
| 8 | ECD | na |  |  | 2876 | 1.034 | -0.0734 | No |
| 9 | NDST3 | na |  |  | 2885 | 1.030 | -0.0668 | No |
| 10 | XYLT2 | na |  |  | 2961 | 1.014 | -0.0639 | No |
| 11 | STC2 | na |  |  | 3009 | 1.002 | -0.0595 | No |
| 12 | IRS2 | na |  |  | 3062 | 0.988 | -0.0556 | No |
| 13 | CTH | na |  |  | 3158 | 0.969 | -0.0540 | No |
| 14 | ABCB6 | na |  |  | 3396 | 0.922 | -0.0601 | No |
| 15 | TSTA3 | na |  |  | 3441 | 0.915 | -0.0562 | No |
| 16 | GPC1 | na |  |  | 3618 | 0.882 | -0.0595 | No |
| 17 | RBCK1 | na |  |  | 3630 | 0.880 | -0.0541 | No |
| 18 | LCT | na |  |  | 3793 | 0.849 | -0.0568 | No |
| 19 | PRPS1 | na |  |  | 4047 | 0.804 | -0.0647 | No |
| 20 | CLN6 | na |  |  | 4163 | 0.788 | -0.0653 | No |
| 21 | ANGPTL4 | na |  |  | 4183 | 0.784 | -0.0610 | No |
| 22 | ARTN | na |  |  | 4502 | 0.734 | -0.0727 | No |
| 23 | GOT1 | na |  |  | 4676 | 0.710 | -0.0770 | No |
| 24 | CLDN9 | na |  |  | 4687 | 0.709 | -0.0727 | No |
| 25 | COL5A1 | na |  |  | 4746 | 0.696 | -0.0710 | No |
| 26 | MED24 | na |  |  | 4824 | 0.683 | -0.0704 | No |
| 27 | PAXIP1 | na |  |  | 4921 | 0.665 | -0.0710 | No |
| 28 | CHST2 | na |  |  | 5270 | 0.610 | -0.0851 | No |
| 29 | CD44 | na |  |  | 5293 | 0.607 | -0.0821 | No |
| 30 | B3GALT6 | na |  |  | 5471 | 0.580 | -0.0875 | No |
| 31 | IDUA | na |  |  | 5557 | 0.570 | -0.0881 | No |
| 32 | PKP2 | na |  |  | 5661 | 0.553 | -0.0897 | No |
| 33 | HOMER1 | na |  |  | 6011 | 0.501 | -0.1047 | No |
| 34 | COG2 | na |  |  | 6146 | 0.483 | -0.1084 | No |
| 35 | NDUFV3 | na |  |  | 6801 | 0.396 | -0.1401 | No |
| 36 | CACNA1H | na |  |  | 6843 | 0.391 | -0.1396 | No |
| 37 | SLC25A13 | na |  |  | 6925 | 0.382 | -0.1413 | No |
| 38 | ME1 | na |  |  | 7136 | 0.354 | -0.1499 | No |
| 39 | B4GALT7 | na |  |  | 7175 | 0.348 | -0.1495 | No |
| 40 | B3GAT3 | na |  |  | 7215 | 0.341 | -0.1492 | No |
| 41 | HDLBP | na |  |  | 7279 | 0.332 | -0.1503 | No |
| 42 | SLC37A4 | na |  |  | 7422 | 0.314 | -0.1556 | No |
| 43 | VCAN | na |  |  | 7533 | 0.301 | -0.1594 | No |
| 44 | GALK2 | na |  |  | 7773 | 0.267 | -0.1701 | No |
| 45 | KIF20A | na |  |  | 7946 | 0.250 | -0.1775 | No |
| 46 | MERTK | na |  |  | 8571 | 0.178 | -0.2090 | No |
| 47 | GCLC | na |  |  | 8925 | 0.131 | -0.2267 | No |
| 48 | B4GALT2 | na |  |  | 9230 | 0.099 | -0.2420 | No |
| 49 | QSOX1 | na |  |  | 9271 | 0.093 | -0.2435 | No |
| 50 | SLC16A3 | na |  |  | 9503 | 0.075 | -0.2551 | No |
| 51 | PPFIA4 | na |  |  | 9532 | 0.071 | -0.2561 | No |
| 52 | TPBG | na |  |  | 9534 | 0.070 | -0.2557 | No |
| 53 | GOT2 | na |  |  | 9565 | 0.067 | -0.2568 | No |
| 54 | TPST1 | na |  |  | 9662 | 0.053 | -0.2615 | No |
| 55 | G6PD | na |  |  | 10053 | 0.012 | -0.2819 | No |
| 56 | NASP | na |  |  | 10061 | 0.011 | -0.2822 | No |
| 57 | PGLS | na |  |  | 10085 | 0.008 | -0.2833 | No |
| 58 | NANP | na |  |  | 10393 | -0.034 | -0.2992 | No |
| 59 | PFKP | na |  |  | 10539 | -0.041 | -0.3066 | No |
| 60 | CITED2 | na |  |  | 10743 | -0.071 | -0.3168 | No |
| 61 | PGM2 | na |  |  | 10868 | -0.089 | -0.3227 | No |
| 62 | ALG1 | na |  |  | 10913 | -0.096 | -0.3243 | No |
| 63 | GUSB | na |  |  | 10994 | -0.105 | -0.3278 | No |
| 64 | SLC35A3 | na |  |  | 10999 | -0.106 | -0.3273 | No |
| 65 | AURKA | na |  |  | 11282 | -0.134 | -0.3412 | No |
| 66 | GNPDA1 | na |  |  | 11314 | -0.138 | -0.3419 | No |
| 67 | HAX1 | na |  |  | 11542 | -0.167 | -0.3527 | No |
| 68 | FKBP4 | na |  |  | 11609 | -0.176 | -0.3550 | No |
| 69 | PMM2 | na |  |  | 11707 | -0.187 | -0.3588 | No |
| 70 | SDC3 | na |  |  | 11878 | -0.214 | -0.3663 | No |
| 71 | PFKFB1 | na |  |  | 11906 | -0.214 | -0.3662 | No |
| 72 | GPR87 | na |  |  | 11939 | -0.214 | -0.3665 | No |
| 73 | POLR3K | na |  |  | 12035 | -0.222 | -0.3700 | No |
| 74 | ANG | na |  |  | 12069 | -0.228 | -0.3701 | No |
| 75 | ALDH7A1 | na |  |  | 12258 | -0.253 | -0.3783 | No |
| 76 | RARS | na |  |  | 12359 | -0.269 | -0.3817 | No |
| 77 | HS2ST1 | na |  |  | 12765 | -0.326 | -0.4008 | No |
| 78 | B4GALT4 | na |  |  | 12800 | -0.328 | -0.4004 | No |
| 79 | ME2 | na |  |  | 12806 | -0.330 | -0.3984 | No |
| 80 | GALE | na |  |  | 12833 | -0.333 | -0.3975 | No |
| 81 | PYGL | na |  |  | 13001 | -0.341 | -0.4039 | No |
| 82 | PC | na |  |  | 13181 | -0.366 | -0.4109 | No |
| 83 | ZNF292 | na |  |  | 13183 | -0.366 | -0.4084 | No |
| 84 | CHPF2 | na |  |  | 13306 | -0.387 | -0.4122 | No |
| 85 | HMMR | na |  |  | 13307 | -0.387 | -0.4096 | No |
| 86 | SDC1 | na |  |  | 13322 | -0.390 | -0.4077 | No |
| 87 | MET | na |  |  | 13324 | -0.390 | -0.4051 | No |
| 88 | PSMC4 | na |  |  | 13485 | -0.411 | -0.4107 | No |
| 89 | KIF2A | na |  |  | 13613 | -0.427 | -0.4144 | No |
| 90 | GYS1 | na |  |  | 13805 | -0.459 | -0.4214 | No |
| 91 | DEPDC1 | na |  |  | 13857 | -0.468 | -0.4209 | No |
| 92 | SPAG4 | na |  |  | 13998 | -0.489 | -0.4249 | No |
| 93 | CENPA | na |  |  | 14298 | -0.521 | -0.4371 | No |
| 94 | PYGB | na |  |  | 14334 | -0.526 | -0.4353 | No |
| 95 | CHPF | na |  |  | 14415 | -0.540 | -0.4359 | No |
| 96 | NOL3 | na |  |  | 14485 | -0.554 | -0.4357 | No |
| 97 | MDH1 | na |  |  | 14491 | -0.555 | -0.4322 | No |
| 98 | PPP2CB | na |  |  | 14561 | -0.566 | -0.4320 | No |
| 99 | LHPP | na |  |  | 14619 | -0.576 | -0.4311 | No |
| 100 | BIK | na |  |  | 14637 | -0.578 | -0.4280 | No |
| 101 | ENO2 | na |  |  | 14703 | -0.591 | -0.4274 | No |
| 102 | RRAGD | na |  |  | 14803 | -0.604 | -0.4285 | No |
| 103 | CAPN5 | na |  |  | 14854 | -0.612 | -0.4270 | No |
| 104 | GALK1 | na |  |  | 14903 | -0.619 | -0.4253 | No |
| 105 | GMPPA | na |  |  | 14997 | -0.632 | -0.4259 | No |
| 106 | EFNA3 | na |  |  | 15291 | -0.685 | -0.4366 | No |
| 107 | ANKZF1 | na |  |  | 15352 | -0.697 | -0.4351 | No |
| 108 | MDH2 | na |  |  | 15652 | -0.754 | -0.4456 | No |
| 109 | PLOD1 | na |  |  | 15757 | -0.775 | -0.4458 | Yes |
| 110 | B3GNT3 | na |  |  | 15828 | -0.788 | -0.4442 | Yes |
| 111 | SRD5A3 | na |  |  | 15901 | -0.802 | -0.4425 | Yes |
| 112 | HK2 | na |  |  | 15926 | -0.806 | -0.4383 | Yes |
| 113 | CDK1 | na |  |  | 15956 | -0.811 | -0.4343 | Yes |
| 114 | SOX9 | na |  |  | 15982 | -0.815 | -0.4301 | Yes |
| 115 | RPE | na |  |  | 16030 | -0.827 | -0.4269 | Yes |
| 116 | NSDHL | na |  |  | 16044 | -0.831 | -0.4219 | Yes |
| 117 | MPI | na |  |  | 16090 | -0.842 | -0.4186 | Yes |
| 118 | TALDO1 | na |  |  | 16127 | -0.852 | -0.4147 | Yes |
| 119 | EXT2 | na |  |  | 16138 | -0.854 | -0.4094 | Yes |
| 120 | DDIT4 | na |  |  | 16203 | -0.871 | -0.4068 | Yes |
| 121 | AGL | na |  |  | 16306 | -0.900 | -0.4061 | Yes |
| 122 | DLD | na |  |  | 16317 | -0.903 | -0.4005 | Yes |
| 123 | CYB5A | na |  |  | 16333 | -0.907 | -0.3951 | Yes |
| 124 | ALDH9A1 | na |  |  | 16400 | -0.923 | -0.3923 | Yes |
| 125 | BPNT1 | na |  |  | 16493 | -0.945 | -0.3907 | Yes |
| 126 | FUT8 | na |  |  | 16559 | -0.959 | -0.3876 | Yes |
| 127 | ALDOA | na |  |  | 16587 | -0.967 | -0.3825 | Yes |
| 128 | TGFBI | na |  |  | 16713 | -1.002 | -0.3822 | Yes |
| 129 | ELF3 | na |  |  | 16854 | -1.043 | -0.3825 | Yes |
| 130 | TXN | na |  |  | 17008 | -1.092 | -0.3831 | Yes |
| 131 | AKR1A1 | na |  |  | 17027 | -1.098 | -0.3766 | Yes |
| 132 | CXCR4 | na |  |  | 17068 | -1.103 | -0.3712 | Yes |
| 133 | LHX9 | na |  |  | 17071 | -1.106 | -0.3638 | Yes |
| 134 | GNE | na |  |  | 17153 | -1.128 | -0.3604 | Yes |
| 135 | TGFA | na |  |  | 17302 | -1.182 | -0.3601 | Yes |
| 136 | NT5E | na |  |  | 17372 | -1.205 | -0.3555 | Yes |
| 137 | PLOD2 | na |  |  | 17461 | -1.240 | -0.3517 | Yes |
| 138 | GFPT1 | na |  |  | 17506 | -1.262 | -0.3455 | Yes |
| 139 | IL13RA1 | na |  |  | 17613 | -1.300 | -0.3422 | Yes |
| 140 | SDC2 | na |  |  | 17642 | -1.310 | -0.3348 | Yes |
| 141 | STMN1 | na |  |  | 17644 | -1.311 | -0.3259 | Yes |
| 142 | SAP30 | na |  |  | 17712 | -1.340 | -0.3203 | Yes |
| 143 | TPI1 | na |  |  | 17749 | -1.359 | -0.3130 | Yes |
| 144 | HSPA5 | na |  |  | 17766 | -1.367 | -0.3045 | Yes |
| 145 | VLDLR | na |  |  | 17779 | -1.371 | -0.2958 | Yes |
| 146 | ISG20 | na |  |  | 17840 | -1.410 | -0.2894 | Yes |
| 147 | P4HA2 | na |  |  | 17913 | -1.447 | -0.2833 | Yes |
| 148 | B4GALT1 | na |  |  | 18015 | -1.493 | -0.2785 | Yes |
| 149 | ARPP19 | na |  |  | 18042 | -1.508 | -0.2696 | Yes |
| 150 | PGAM1 | na |  |  | 18051 | -1.512 | -0.2598 | Yes |
| 151 | SDHC | na |  |  | 18153 | -1.583 | -0.2543 | Yes |
| 152 | COPB2 | na |  |  | 18316 | -1.697 | -0.2513 | Yes |
| 153 | PAM | na |  |  | 18328 | -1.699 | -0.2403 | Yes |
| 154 | UGP2 | na |  |  | 18385 | -1.741 | -0.2314 | Yes |
| 155 | GLCE | na |  |  | 18564 | -1.885 | -0.2280 | Yes |
| 156 | P4HA1 | na |  |  | 18626 | -1.958 | -0.2179 | Yes |
| 157 | GLRX | na |  |  | 18639 | -1.972 | -0.2051 | Yes |
| 158 | IGFBP3 | na |  |  | 18665 | -2.008 | -0.1928 | Yes |
| 159 | IDH1 | na |  |  | 18690 | -2.035 | -0.1802 | Yes |
| 160 | FAM162A | na |  |  | 18708 | -2.062 | -0.1671 | Yes |
| 161 | AK3 | na |  |  | 18732 | -2.092 | -0.1540 | Yes |
| 162 | AK4 | na |  |  | 18825 | -2.239 | -0.1437 | Yes |
| 163 | PDK3 | na |  |  | 18854 | -2.278 | -0.1296 | Yes |
| 164 | KDELR3 | na |  |  | 18881 | -2.328 | -0.1152 | Yes |
| 165 | STC1 | na |  |  | 18901 | -2.351 | -0.1002 | Yes |
| 166 | SOD1 | na |  |  | 18910 | -2.370 | -0.0845 | Yes |
| 167 | CASP6 | na |  |  | 18952 | -2.470 | -0.0699 | Yes |
| 168 | MXI1 | na |  |  | 18988 | -2.556 | -0.0543 | Yes |
| 169 | EGLN3 | na |  |  | 19047 | -2.739 | -0.0388 | Yes |
| 170 | PGK1 | na |  |  | 19154 | -3.315 | -0.0218 | Yes |
| 171 | DSC2 | na |  |  | 19172 | -3.540 | 0.0014 | Yes |
Table: GSEA details [plain text format]

  

Fig 2: HALLMARK\_GLYCOLYSIS      
 Blue-Pink O' Gram in the Space of the Analyzed GeneSet

  

Fig 3: HALLMARK\_GLYCOLYSIS: Random ES distribution      
 Gene set null distribution of ES for **HALLMARK\_GLYCOLYSIS**
